# Continuing medical education in Brazil: what about obstetricians and gynecologists?

**DOI:** 10.1590/S1516-31802005000100002

**Published:** 2005-01-02

**Authors:** Nelson Sass, Maria Regina Torloni, Bernardo Garcia de Oliveira Soares, Álvaro Nagib Atallah

**Keywords:** Continuing medical education, Obstetrics, Gynecology, Evidence-based medicine, Medical education, Educação médica continuada, Ginecologia, Obstetrícia, Medicina baseada em evidências, Educação médica

## Abstract

**CONTEXT:**

In Brazil, obstetricians and gynecologists are not required to submit to periodical evaluations to ascertain their professional competence in dealing with new concepts and therapies.

**OBJECTIVES:**

To evaluate the performance of a group of obstetricians and gynecologists on a written evidence-based obstetrics test and determine their opinions and use of systematic reviews.

**TYPE OF STUDY:**

Prospective cohort.

**SETTING:**

Brazilian Obstetrics and Gynecology Congress 2001.

**METHODS:**

230 doctors agreed to participate in the study during a national obstetrics and gynecology congress. Participants took an individual anonymous written multiple-choice test with seven questions on clinical obstetrics, one question on the interpretation of a meta-analysis graph and two questions on their opinions and actual use of systematic reviews. Scores were analyzed and compared after grouping the participants according to year of graduation, residence training, doctoral program and faculty status.

**RESULTS:**

The general average score was 49.2 ± 17.4. The scores tended to decline as the years since graduation advanced. Doctors who graduated in the last five years had higher scores than those who graduated over 25 years ago (52.2 versus 42.9). The performance did not vary according to medical residence, postgraduate program or teaching status. While 98.2% considered systematic reviews relevant, only 54.9% said that they routinely used this source of information.

**DISCUSSION:**

The participants' average score was low, even though they were highly qualified and trained. Despite the limitations of the study, the results are worrisome. If motivated physicians participating in a national congress obtained such low scores, we can speculate that the results might be even worse among other doctors that do not attend these events.

**CONCLUSIONS:**

These findings suggest that Brazilian obstetricians and gynecologists could benefit from continuing medical education and raise questions about the recycling methods currently available.

## RESUMO

**CONTEXTO:**

A educação médica continuada definida de forma genérica como todas as formas pelas quais os médicos aprendem após a sua formação, é um assunto de interesse tanto dos médicos como dos pacientes em geral. No Brasil, os tocoginecologistas não são obrigados a se submeter a exames periódicos para certificar sua competência e atualização profissional, o que torna a educação médica continuada sujeita à de consciência pessoal.

**OBJETIVOS:**

Avaliar o desempenho de um grupo de tocoginecologistas em um teste escrito com perguntas sobre obstetrícia baseada em evidências e determinar sua opinião e uso regular de revisões sistemáticas.

**TIPO DE ESTUDO:**

Prospectivo de coorte.

**LOCAL DO ESTUDO:**

Congresso Brasileiro de Obstetrícia e Ginecologia 2001, em São Paulo.

**MÉTODOS:**

230 médicos participaram voluntariamente do estudo durante o Congresso. Todos preencheram um questionário escrito, anônimo, individual com sete perguntas de múltipla escolha sobre condutas obstétricas clínicas baseadas em evidência, uma pergunta sobre interpretação de um gráfico de metanálise e duas perguntas sobre opinião e uso de revisões sistemáticas. Foi calculada a porcentagem de acerto de todos os participantes nas perguntas de múltipla escolha. Os resultados foram também analisados conforme o tempo de formado, residência, pós-graduação e docência.

**RESULTADOS:**

A média geral foi 49,2 + 17,4. As notas tenderam a cair com o tempo decorrido desde a formatura. Os médicos formados nos últimos cinco anos tiveram notas maiores que aqueles formados há mais de 25 anos (52,2 *versus* 42,9). O desempenho não variou significativamente conforme ter ou não residência, pós-graduação ou ser docente. Enquanto 98,2% consideravam as revisões sistemáticas relevantes, apenas 54,9% apontou o uso regular dessa fonte de informação médica.

**DISCUSSÃO:**

A nota média dos participantes foi baixa, apesar de sua boa formação e qualificação. Apesar das limitações deste estudo, os resultados são preocupantes. Se médicos motivados que participavam de um congresso nacional tiveram notas tão baixas, podemos especular que os resultados seriam ainda piores entre outros colegas que não participam desses eventos.

**CONCLUSÃO:**

Esses achados sugerem que os tocoginecologistas brasileiros poderiam se beneficiar com educação médica continuada e levanta questões acerca das formas atuais de reciclagem médica.

## INTRODUCTION

Perhaps one of the most important and delicate questions of medical practice is how to ensure that, years after the completion of their formal training, medical doctors maintain and improve their problem-solving skills and technical competence, and also keep up with the advances in their field of medicine.

Worldwide, as patients become more educated and informed about health issues in general and their particular condition, they also become more demanding consumers. This changing attitude, along with the constant influx of new medical information, has greatly changed physicians' working conditions, thereby making continuing medical education (CME) a obligatory part of every doctor's life.

CME is defined as "any and all the ways by which doctors learn after formal completion of their training".^1^ The main objective of CME is to maintain and improve physicians' performance years after graduation and this is a common point of interest for doctors and patients alike, as well as a public health concern.

Several countries have passed laws that condition the revalidation of medical licenses to periodic assessment of medical expertise.^2^ In Brazil, due to concerns about the quality of medical schools, the federal government has instituted annual examinations for all final year medical students, which began in 1996.^3^ However, after graduation, Brazilian doctors are not periodically evaluated in their specialties to ascertain their professional competence in dealing with new concepts and therapies, thus making continuing medical education a matter of personal conscience.

Over the last few years, the theoretical basis and teaching methods traditionally employed in CME have dramatically changed. And despite some controversy regarding the best forms of intervention, systematic reviews available in the literature emphasize that educational processes can indeed modify clinical practice.^4-7^ These studies suggest that, in the medical profession, interactive meetings and educational activities relating to daily practice are the most efficient forms of learning.

Unfortunately, while the organizing committees of symposiums, meetings and congresses devote much time and attention to programming and promoting successful events, they seldom worry about educational questions such as: Are the included topics and presentations relevant and consistent enough to modify the clinical practice of participating physicians? Is it possible to periodically check the level of knowledge of the attending specialists? Is there a way of doing this without embarrassing the participants?

There are few publications about these educational questions, possibly because of their delicate nature. Many authors also consider this to be a very specific and narrow field of interest and others claim it is diffi-cult to objectively evaluate these questions. Criticism of the methodology employed in these studies is valid, since it is almost impossible to have control groups (not exposed to information) and therefore to organize randomized trials.

In the hope we might contribute to these important and delicate questions, we decided to evaluate, during a national congress, the performance of Brazilian physicians in a written test containing clinical questions relevant to contemporary obstetric practice.

The objective of this study was to evaluate the performance of a group of doctors participating in a national obstetrics and gynecology congress who spontaneously agreed to submit to a written evidence-based obstetrics test. A secondary objective was to determine the participants' opinions and actual use of systematic reviews.

## METHODS

This was a prospective cohort study involving a written test voluntarily taken by a group of doctors attending the 49^th^ Brazilian Congress of Obstetrics and Gynecology, which was held in the city of São Paulo, Brazil, in November 2001.

The authors handed out a test-sheet to all doctors visiting the Cochrane stand and to those attending the opening of the "Evidencebased obstetrics and gynecology course" held during the Congress. The subjects were invited to fill out and immediately hand back the test, as a voluntary collaboration.

The test was anonymous, but had an identification section that asked about the year and place (state and country) of graduation and whether the participant had undergone residency training in obstetrics and gynecology, had acquired postgraduate education, or was teaching in a formal medical program. This was followed by two questions about their use of and interest in systematic reviews and, finally, seven multiple-choice questions related to obstetric practice and one multiple-choice question on the interpretation of a metaanalysis graph. All the multiple-choice questions had only one right answer (and three wrong answers).

The questions were:

1. In your practice, do you use systematic reviews or meta-analysis as a source of information?
2. Do you consider systematic reviews relevant for obtaining information?
3. At what gestational age is the use of corticosteroids considered beneficial?
4. If you could only order one ultrasound during routine prenatal care, which gestational age would you pick?
5. Which of these interventions could be effective in a pregnant patient with a previous history of severe pre-eclampsia?
6. What delivery route would be best for a patient with three previous vaginal deliveries whose fetus was full-term, weighed 3,000 g and was clearly in the breech presentation?
7. In which of the following situations associated with prematurity would you use corticosteroids?
8. Which of the following anti-convulsive medications would you prescribe for a patient with eclampsia?
9. Which of the following medications would you not recommend for a 15-week pregnant woman with a blood pressure of 150 × 105?
10. Which of the results presented in the meta-analysis graph below do you consider beneficial for use in or for changing your clinical practice?

The correct answers to questions 3 to 10 were based on the results of systematic reviews of randomized controlled trials available in the Cochrane Library.^8-17^ The score was calculated by dividing 100 by 8, with possible scores of 0, 12.5, 25, 37.5, 50, 62.5, 75, 87.5 or 100, according to the number of correct answers: respectively 0, 1, 2, 3, 4, 5, 6, 7 or 8. Questions unanswered (blank) were considered wrong.

The results (average score) for the whole group were calculated and then the participants were divided into six different categories according to the number of years elapsed since graduation, in five-year intervals (0-5, 6-10, 11-15, 16-20, 21-25, 26 or more) and the average score for each category was calculated and compared, using the first group (graduated in the last five years) as reference. We also compared the average scores of those with and without residence training, postgraduate education and teaching status. Finally, we compared the scores of those who routinely used systematic reviews with the scores of non-users.

The results were tabulated as percentages, means and standard deviations and were compared using variance analysis (ANOVA) and the chi-squared and Fisher exact tests. p < 0.05 was considered significant.

## RESULTS

The study population consisted of 230 doctors, representing 4.6% of the 4,975 physicians registered in the congress. Of the participants, 63 were recruited while visiting the Cochrane stand. There was no record of the total number of visitors to the stand during the three days of the event. Approximately one third (167 or 35.6%) of the 469 students attending the course volunteered to participate in the study. All participants adhered spontaneously to the study. Since there were no requirements or rewards for participation in the study, all participants were self-selected.

The length of time since graduation ranged from 9 months to 43 years, with a mean of 15.2 years ± 9.8 (standard deviation). Most of the group (61.2%) had graduated in the southeastern region of Brazil, which was also the location of the event. The majority (85.2%) had concluded medical residence, 37.4% had postgraduate degrees (MSc or PhD) and 13.9% were faculty members, teaching in medical schools. A large number of participants did not answer the questions related to postgraduation and teaching (Table 1).

**Table 1:** Characteristics of the 230 Brazilian obstetrics and gynecology participants in a cohort on the use of medical evidence provided by meta-analysis

	n	%
Total congress enrollment	4,975	100.0
Survey participants	230	4.6
- Recruited in the evidence-based course	167	72.6
- Recruited at the Cochrane stand	63	27.4
Place of graduation (regions)		
Southeast	134	58.3
Northeast	46	20.0
South	21	9.1
North	11	4.8
Center-west	7	3.0
Blank answer	11	4.8
Length of time since graduation (years)		
1 to 5	40	17.4
6 to 10	30	13.0
11 to 15	40	17.4
16 to 20	32	13.9
21 to 25	41	17.8
26 or more	37	16.1
Blank answer	10	4.4
Medical residence		
Yes	196	85.2
No	22	9.6
Blank answer	12	5.2
Postgraduate program		
Yes	86	37.4
No	82	35.6
Blank answer	62	27.0
Faculty teaching		
Yes	32	13.9
No	74	32.2
Blank answer	124	53.9

More than half (54.9%) indicated that they routinely used systematic reviews (question 1) and 98.2% considered this to be a relevant source of information (question 2). The use of systematic reviews declined as the time since graduation elapsed. Their use was significantly higher among doctors who had graduated in the last 5 years than among those who had graduated over 25 years ago (67.5% versus 35.1%, p = 0.004).

**Figure 1:**
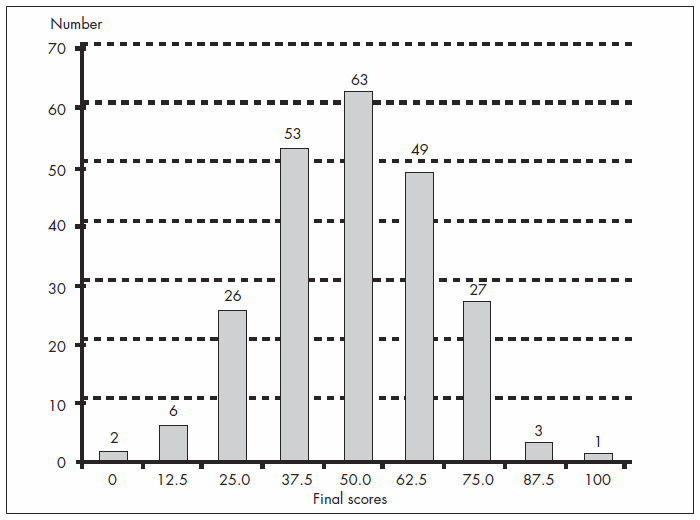
Distribution of final scores for the 230 participants in a cohort on the use of medical evidence provided by meta-analysis.

**Figure 2:**
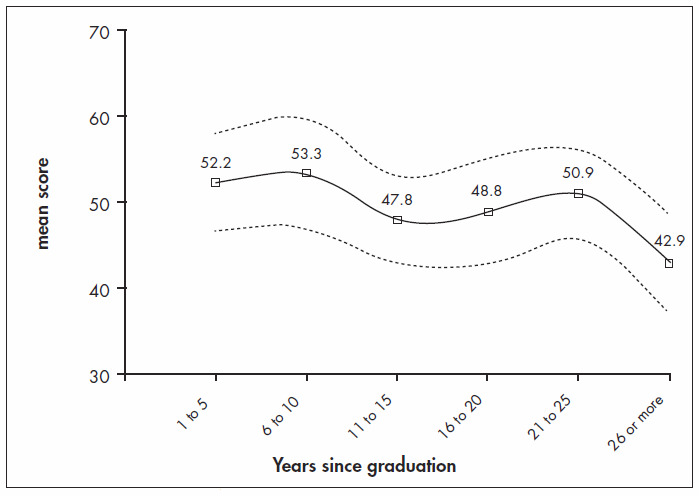
Mean scores and standard deviation of performance in a written evidencebased test of medical doctors, according to time elapsed since graduation.

The general performance of the group was low (Figure 1), with a mean score of 49.2 (± 17.4). Analysis of the results, grouped in the six categories according to the time since graduation (five-year intervals), revealed progressively lower mean score, thus suggesting a descending slope as time elapsed after finishing medical school (Figure 2). The average score for the recently graduated doctors (52.2 ± 18.5) was higher than for the oldest physicians (42.9 ± 17.1), although this difference did not reach significance (p = 0,14). The performances of those with or without residence (mean score: 50.1 ± 17.3 versus 44.9 ± 17.1; p = 0.179), with or without postgraduate education (50.4 ± 18.9 versus 50.3 ± 15.9; p = 0.963) and with or without teaching status (53.5 ± 16.7 versus 52.7 ± 19.9; p = 0.829) were similar.

With regard to the specific questions (Table 2), the one about the best anticonvulsive medication for eclampsia (question 8) had the highest proportion of correct answers (90.4%), followed by the interpretation of the meta-analysis graph (question 10), with 67%. Question 10 also had the highest percentage of blank answers (12.6%). The use of antenatal steroids also had statistically more correct answers (64.8%). The question about the prevention of pre-eclampsia (question 5) had the lowest scores, with only 12.6% of the participants indicating the correct answer (calcium). The questions about delivery route for a breech presentation and gestational age for ultrasound also had poor results, with only 25.7% and 39.6% of answers correct, respectively. The questions concerning contraindications for the use of steroids and anti-hypertensive medication in the second trimester had equal proportions of right and wrong answers. Doctors who graduated in the last 5 to 10 years had the highest percentage of correct answers (40%, p = 0.034) in the question about the prevention of pre-eclampsia, in comparison with the other five categories. No significant differences in the percentages of correct answers obtained by the six categories of length of time since graduation were observed for the other questions.

**Table 2:** Specific distribution of answers to 8 questions by gynecologists and obstetricians in a medical congress in São Paulo, 2001

Question number	Subject	Right		Wrong		p*	Blank	
		n	%	n	%		n	%
3	antenatal steroids	149	64.8	80	34.8	< 0.001	1	0.4
4	gestational age for ultrasound	91	39.6	133	57.8	< 0.001	6	2.6
5	prevention of pre-eclampsia	29	12.6	190	82.6	< 0.001	11	4.8
6	breech delivery	59	25.7	170	73.9	< 0.001	1	0.4
7	contra-indications for steroids	108	47.0	112	48.7	0.709	10	4.3
8	anticonvulsants for eclampsia	208	90.4	22	9.6	< 0.001	0	–
9	drugs for hypertension	109	47.4	117	50.9	0.456	4	1.7
10	interpretation of meta-analysis	154	67.0	47	20.4	< 0.001	29	12.6

* *Comparing proportions of right and wrong answers.*

**Figure 3:**
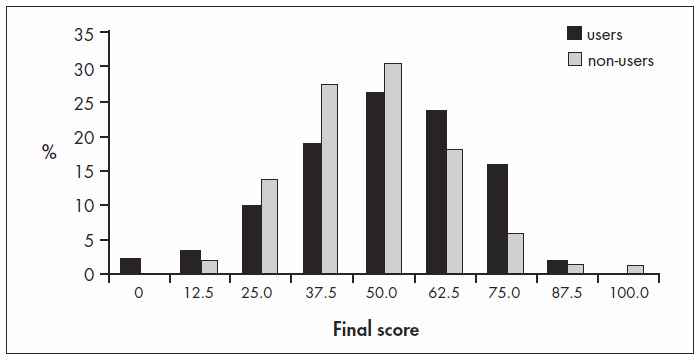
Score distribution for users and non-users of systematic reviews among doctors answering a written test in a medical congress.

Comparing the distribution of scores for the 123 "users" of systematic reviews and the 101 "non-users", we could not detect a significant difference, but there seemed to be a possible trend towards higher scores in the first group (Figure 3).

## DISCUSSION

The Brazilian Congress of Obstetrics and Gynecology is held every two years and is considered to be the most important Brazilian event in its field. It covers a large range of themes organized into talks, panel discussions and laboratory-sponsored symposiums. Evidently, one of the goals of this event is to provide new and relevant information, so as to keep practicing obstetricians and gynecologists up to date in their field of work. Considering that, every month, the average Brazilian doctor's mailbox is stuffed with folders, invitations and advertisements about meetings, events and courses, it is logical to assume that most physicians probably participate in other educational activities held in between the national congresses.

Yet, the participants' mean scores were low, with only 34.8% (80/230) of the participants having a score above 50.0. These results make us wonder whether Brazilian obstetricians and gynecologists really receive the best evidence in the field of obstetrics, and whether this information is offered in an efficient way that is capable of modifying professional practice.

We feel it is wise to interpret the results of this study with caution, since participation was spontaneous and voluntary and the subjects were not randomly selected or meant to be representative of the population of Brazilian obstetricians and gynecologists. Volunteer samples and studies on self-reported rather than observed practice are always subject to criticism. On the other hand, since most participants were probably sincerely interested in the latest therapeutic advances and solid evidence (since they were enrolled in an evidence-based course or were visiting the Cochrane stand), we would perhaps expect a better performance by these individuals in the test. Even more worryingly, if motivated physicians participating in a national congress obtained such low scores, we can speculate that the results might be even worse among the other Brazilian obstetricians and gynecologists that do not attend such events.

The general performance of the physicians in the test was average, and tended to worsen as the years since graduation advanced. In fact, younger doctors demonstrated more interest in and use of systematic reviews, but this might not be the only explanation, since we did not observe significant differences between the scores of users and non-users of systematic reviews (Figure 3). Younger physicians possibly scored higher because of the more up-to-date information that they had received during their recent years of schooling and residency training. However, another troubling observation was that medical residence, postgraduate education or teaching status did not ensure better performance in the test.

Perhaps one of the greatest challenges of medical practice is to keep a certain balance between growing professional competence and confidence as the years of clinical experience grow and, at the same time, to keep a certain flexibility and humility in order to adjust to new concepts and interventions, which are sometimes completely different from those learned while in medical school.^7^

In Brazil, in order to be recognized as a specialist in a specific field of medicine, doctors have to pass a difficult written examination with many complex and detailed questions ranging from anatomy and physiology to chemotherapy schemes. These examinations are generally taken by young physicians, soon after the conclusion of their residence and are seen as rituals of entry to a new phase of their medical career. However, after this initial examination, most specialty boards do not require periodic re-certification or verification of doctors' competence, or documentation of their efforts to keep updated in the face of the constant input of new and important scientific information that is essential for ensuring good clinical practice.

While each professional has a personal responsibility to keep up with the advances within his specialty, we cannot dismiss the responsibility of various institutions and specialty boards for ensuring that their members undergo periodic refreshers.

We were surprised and worried that so many well-trained physicians (> 80% had completed medical residence) gave wrong answers to simple clinical questions that probably appear on a daily basis in a busy obstetrics practice. These results make us ponder about the way medicine is taught, practiced and regulated.

Considering the inexorable tendency of medical practice to be based on solid evidence, we believe that doctors involved with medical education should rely on efficient teaching methods that are clearly capable of modifying clinical practice.^6^

Continuing medical education is constantly improving, in part due to the growing use of evidence-based medicine and ease of obtaining this information. Curiously, in spite of the almost unanimous (98.2%) belief that systematic reviews are relevant, only half of the participants actually use meta-analyses as a source of information, and their use is more frequent among the recently graduated. This may be related to the way medicine is traditionally taught or practiced in most hospitals, where the authority or opinion of the chief professor or head physician can be taken as final and unquestioned. There is little room in most traditional Brazilian institutions for this new form of medical information, and a considerable number of doctors simply do not know how to access it. Fortunately, in Brazil the Cochrane Collection is now easily available using any computer linked to the internet (www.bireme.br/cochrane), and efforts to train more physicians on its use would undoubtedly be beneficial. In fact, 20.4% of the participants answered the question about the interpretation of a meta-analysis graph incorrectly, and another 12.6% left the question blank (Table 2), thus suggesting difficulty in dealing with this kind of information.

Our analysis of the test scores had no intention of measuring the level of knowledge of Brazilian obstetricians, and should not be taken as a judgement of their competence or expertise. However, these results may serve as the basis for further studies and debates on the need for continuing medical education and on the efficacy of traditional recycling methods, which is a matter of concern for physicians and society.

## CONCLUSIONS

The participants' average score was low, despite their high qualifications and training. Almost all participating physicians considered systematic reviews to be relevant but only half were in fact using them regularly as a source of information. Doctors who had graduated in the last five years used more systematic reviews and had higher average scores than those who graduated 26 or more years ago. These findings suggest that Brazilian obstetricians and gynecologists could benefit from continuing medical education and question the refresher methods currently employed.
